# Organic cultivation of Ashwagandha with improved biomass and high content of active Withanolides: Use of Vermicompost

**DOI:** 10.1371/journal.pone.0194314

**Published:** 2018-04-16

**Authors:** Amandeep Kaur, Baldev Singh, Puja Ohri, Jia Wang, Renu Wadhwa, Sunil C. Kaul, Pratap Kumar Pati, Arvinder Kaur

**Affiliations:** 1 Department of Zoology, Guru Nanak Dev University, Amritsar, Punjab, India; 2 Department of Biotechnology, Guru Nanak Dev University, Amritsar, Punjab, India; 3 Drug Discovery and Assets Innovation Lab, DBT-AIST International Laboratory for Advanced Biomedicine (DAILAB), National Institute of Advanced Industrial Science & Technology (AIST), Tsukuba, Japan; Indian Institute of Technology Delhi, INDIA

## Abstract

*Withania somnifera* (Ashwagandha) has recently been studied extensively for its health-supplementing and therapeutic activities against a variety of ailments. Several independent studies have experimentally demonstrated pharmaceutical potential of its active Withanolides, Withaferin A (Wi-A), Withanone (Wi-N) and Withanolide A (Wil-A). However, to promote its use in herbal industry, an environmentally sustainable cultivation and high yield are warranted. In modern agriculture strategies, there has been indiscriminate use of chemical fertilizers to boost the crop-yield, however the practice largely ignored its adverse effect on the quality of soil and the environment. In view of these, we attempted to recruit Vermicompost (Vcom, 20–100%) as an organic fertilizer of choice during the sowing and growing phases of Ashwagandha plants. We report that (i) pre-soaking of seeds for 12 h in Vermicompost leachate (Vcom-L) and Vermicompost tea (Vcom-T) led to higher germination, (ii) binary combination of pre-soaking of seeds and cultivation in Vcom (up to 80%) resulted in further improvement both in germination and seedling growth, (iii) cultivated plants in the presence of Vcom+Vcom-L showed higher leaf and root mass, earlier onset of flowering and fruiting and (iv) leaves from the Vcom+Vcom-L cultivated plants showed higher level of active Withanolides, Withanone (Wi-N), Withanolide A (Wil-A) and Withaferin A (Wi-A) and showed anticancer activities in cell culture assays. Taken together, we report a simple and inexpensive method for improving the yield and pharmaceutical components of Ashwagandha leaves.

## Introduction

*Withania somnifera*, a highly reputed plant in Ayurveda, has been traditionally used in India and its neighbouring countries as a home remedy and dietary supplement for thousands of years. It is widely distributed in the tropical climate of Indian sub-continent, South Africa, the Mediterranean and Middle East regions. A well described pharmacological profile of *W*. *somnifera* indicates a wide array of therapeutic properties including anti-inflammatory, anti-microbial, anti-oxidant, adaptogenic, aphrodisiac, analgesic, anti-arthritic, anti-stress, neurological, immunostimulant, cardio-protective and anticancer effects [1–7]. Several of these effects have been attributed to its secondary metabolites such as alkaloids, steroidal lactones, steroids, flavonoids, glycowithanolides and saponins [8–10]. Although all parts of the plant including stem, leaves, roots, flowers, seeds and bark possess these constituents; they particularly accumulate in roots and leaves [11]. Recently, it has been shown that as compared to the roots, leaves possess higher content of Withaferin A and Withanone [12].

*W*. *somnifera* is cultivated for food-supplement, herbal and medicine industries. With growing laboratory evidence of its medicinal value for a variety of ailments, sustained supply of active ingredient-enriched plants is desirable. In this context, there is a need for exploring various strategies for its simple, inexpensive and eco-friendly cultivation. Although, *W*. *somnifera* grows on sandy loam and well-drained soil, its cultivation faces serious challenges due to low seed germination and seed viability, a long period between planting and harvesting and low yield [13]. Hence, scientific intervention is required to enhance seed germination, plant biomass and its pharmaceutically active constituents.

Studies in the past have reported that pre-treatment of seeds with KNO_3,_/KH_2_PO_4_/NaNO_3_/PEG improved seed germination of a variety of plants [14–16]. Similarly, various mineral nutrients supplemented in the soil are also known to have bearing on plant biomass [17]. However, adding synthetic fertilizer to boost plant growth might increase cultivation cost and bring undesirable effects on the ecosystem [18]. Furthermore, prolonged use of synthetic fertilizers changes microbial diversity and chemical properties of soil, causes acidification of soil and reduces uptake of nutrients by plant [19,20]. However, fortification of soil with Vermicompost (Vcom) has been widely accepted as an excellent organic amendment for cultivation of several important crops [21–23]. Vcom is nutrient rich organic material prepared by the combined action of earthworms and microorganisms on the organic wastes [24]. It naturally provides organic nutrients and enormous humic substances as well as nitrates, phosphates, exchangeable calcium, potassium, sulphur, magnesium and other micronutrients required for plant growth [25]. It possesses high porosity, aeration, drainage and water holding capacity [26]. Furthermore, Vermicompost leachate (Vcom-L) and Vermicompost tea (Vcom-T), the liquid products of vermicomposting, containing humified organic matter and nutrients in soluble form are also known to have stimulating effect on seed germination and growth of plants [27,28].

Knowing the favourable attributes of Vcom and its liquid products, we aimed to analyze the effect of Vcom, Vcom-L and Vcom-T on seed germination, growth parameters, onset of maturity (flowering and fruiting) and its bearing on withanolides contents on *W*. *somnifera*. To best of our knowledge, there is no report on propagation and evaluation of important phytochemicals of a high valued medicinal plants such as *W*. *somnifera* using Vcom and its associated products. The present study proposes a scientifically rational and environmentally sustainable propagation system that could be extended to many other medicinal plants in future.

## Materials and methods

### Preparation of Vermicompost (Vcom), Vermicompost tea (Vcom-T) and Vermicompost leachate (Vcom-L)

Young non clitellate *Eisenia fetida*, collected from the vermifarm of Guru Nanak Dev University (GNDU), Amritsar (http://www.gndu.ac.in) were used for the preparation of Vcom, Vcom-T and Vcom-L. *E*. *fetida*, an epigeic polychaete, is commonly used for vermicomposting because it is hardy, prolific breeder and feeds on a variety of organic matter. Vcom was prepared by processing cattle dung (collected from dairy farms in the vicinity of GNDU, Amritsar) for 45 days in windrows at 27±2°C temperature and 70–80% moisture. Vcom-L was prepared from a mixture of cattle dung, vegetable peels (collected from hostel mess, GNDU, Amritsar) and dry leaf litter (collected from the lawns of GNDU, Amritsar) in the ratio of 2:1:1 (w/w) in a stainless steel reactor (S1 Fig). The leachate was collected on alternate days for two months and stored at 4°C. It was brought to room temperature before use. For preparation of Vcom-T, Vcom was mixed in distilled water in the ratio of 1:10 (w/v). The mixture was stirred on a magnetic stirrer at 40°C for 12 h and filtered through filter paper before use.

### Plant material and treatments

Seeds of *W*. *somnifera* were collected from 2-years old plants (from the experimental plots at GNDU, Amritsar), washed with distilled water, air dried and divided in two groups. Before sowing, the seeds were separately given pre-soaking treatments for 1 h (Group-I) and 12 h (Group-II) in distilled water (DW), a saturated solution of KNO_3_ (KN), Vcom-L and Vcom-T. Different percentages of Vcom (0, 20, 40, 60, 80 and 100%; w/w) were mixed in the potting mixture which contained 1:1 ratio of sand: loamy soil and used for subsequent experimentation. The elemental composition of plant growth substrates was evaluated using protocol published earlier [29,30].

### Germination and plant growth study

Experiment was performed in a completely randomised design (CRD), under greenhouse conditions with 12 h photoperiod and 25±2°C temperature (day/night). Twenty seeds from each soaking treatment were sown separately in earthen pots (10-cm diameter containing 160 g potting mixture) having variable Vcom (%) in quadruplicate. Thereafter, pots were watered on alternate days to maintain adequate moisture (50–60%) for germination and seedling growth. Germination (sprouting of cotyledonary leaves) was recorded after fifteen days of sowing. After forty five days of sowing, three seedlings were randomly selected from each replication for recording shoot length (cm), shoot diameter (cm), root length (cm), leaf number, root number, and total biomass (g/seedling). Seedlings of uniform size (with best growth characteristics) were transplanted to larger earthen pots (25 cm diameter having 2 kg prepared potting mixtures) at the rate of one seedling per pot in triplicate and divided into Vcom (Vcom-L not supplied) and Vcom-L (Vcom+Vcom-L) groups. The plants were irrigated with tap water on alternate days, Vcom-L group was treated with 200 ml of Vcom-L per pot.

Growth parameters such as shoot length (cm), shoot diameter (cm), number of branches/plant, number of leaves/plant and mean leaf area (cm^2^) were recorded sixty days after transplantation, while fresh and dry weight (g/plant) was determined at the end of the experiment (seventy five days after transplantation). For measuring leaf area, fully expanded young leaves (third and fourth from top) were excised at the base and immediately digitally photographed (Nikon, Coolpix S2700 camera, Nikon Corp., Japan). Images were then analysed using ImageJ^®^ software version 1.4 [31]. Shoot and root parts of the plant were separated, washed with water, blotted with filter paper and weighed to determine the fresh weight. Dry weight was determined after oven-drying the shoot and root at 75°C until a constant weight was achieved. During the experiment, plants were also monitored for the number of days taken for onset of flowering and fruiting. The experiment was repeated twice to validate the results.

### Preparation of methanolic extract of leaves

One gram dry leaf powder from plants grown under the conditions as mentioned was mixed with 10 ml methanol and sonicated for 45 min at room temperature (25± 2°C). The supernatant was filtered through Whatman No. 1 filter paper. The residue was extracted twice with same volume of methanol. The pooled filtrate was then vacuum-dried on a BȔCHI rotary-evaporator (consisting of rota vapour R-210, re-circulating chiller B-740, heating bath B-491 and vacuum pump V-7100, BȔCHI Ltd., Flawil Switzerland) at 40°C. Residues were reconstituted in methanol (10 ml), filtered through 0.22 μm filter and used for the analysis of Withanolides.

### High-performance liquid chromatography (HPLC) analysis

Withanolides (Withaferin A: Wi-A, Withanolide A: Wil-A and Withanone: Wi-N) were quantified using HPLC (Agilent 1260 infinity series HPLC; Agilent technologies, Santa Clara, CA), consisting of Quaternary Pump VL (G1311C) and degasser, standard auto sampler (G1329B ALS) and diode array detector (G1315D DAD VL). Separations were carried on Agilent ZORBAX Eclipse C18 column (4 X 10 mm). An isocratic elution was carried out using water (Solvent A) and methanol (Solvent B) in the ratio of 40:60 (v/v) with a run time of 40 min. The injection volume was 10 μl and the eluents were detected at 237 nm. Calibration curves were prepared from the purified standards of Withanolides [Wi-A (LOD = 4.086 ng/ml; LOQ = 12.38 ng/ml), Wil-A (LOD = 5.096 ng/ml; LOQ = 15.44 ng/ml) and Wi-N (LOD = 1.722 ng/ml; LOQ = 5.742 ng/ml] and the results were expressed in mg/g dry weight of the sample.

### Preparation of β-cyclodextrin-assisted water extract of Ashwagandha leaves

Water extract (10% w/v) was prepared from dried powder of leaves grown under different conditions as indicated. For cyclodextrin (CD)-assisted aqueous extraction, the dried leaf powder (10% w/v) was mixed with aqueous solution of β-CD (2%). The mixture was stirred for 24 h at 37°C with slow shaking (90 rpm) in TAITEC Bio-Shaker BR-43FL. The slurry was centrifuged at 3500 rpm for 10 min and the supernatant was filtered through 0.45-micron filter. The filtrate was called β-CD extract. The precipitate was dissolved in DMSO as described [12] and called β-DM extract. CD and DM extracts obtained from dry leaf powder (10% by weight) were considered 100% and added to the cell culture medium in a range of 0.05~1%, corresponding to 50 μg—1 mg/ml of dried leaf powder.

### *In vitro* cytotoxicity assay

Human normal fibroblasts (TIG-3) were procured from Health Science Research Resources Bank, Japan. Osteosarcoma (U2OS) cell lines were obtained from DS Pharma, Japan, and cultured in Dulbecco’s Modified Eagle’s Medium DMEM (Invitrogen)-supplemented with 10% fetal bovine serum in a humidified incubator (37°C and 5% CO_2_). Cells were cultured at 40–60% confluency and treated with different kinds of extracts as indicated. Cells were incubated at 37°C for 36–48 h, following which cytotoxicity was estimated by MTT {3-(4,5-dimethylthiazol-2-yl)-2, 5-diphenyltetrazolium bromide} assay (Life Technologies) as described earlier [5].

### Statistical analyses

Experimental data were subjected to two-way ANalysis of VAriance (ANOVA) test to analyze the correlation within Vcom and pre-soaking groups and the difference between means of different Vcom proportions (%; taking untreated 0% group as control) and pre-soaking treatments was compared by Tukey’s HSD at p≤0.05 (5% level of significance) using SigmaStat version 3.5 (SystatSoftware Inc.).

## Results

### Effect of pre-soaking and Vcom treatments on germination and seedling growth

**Fig 1:**
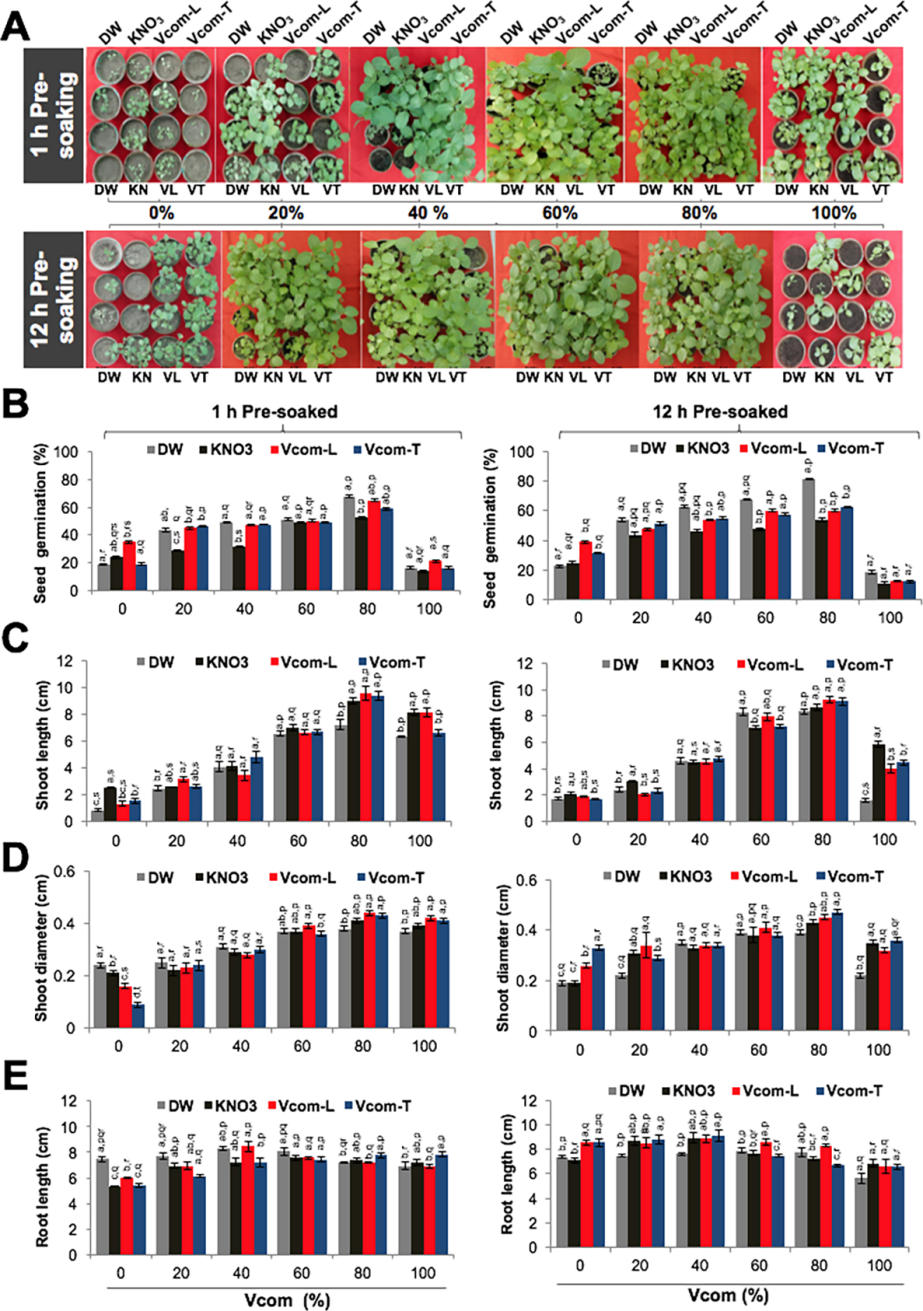
Effect of pre-soaking on *W*. *somnifera* growth. Figure showing the effect of pre-soaking and Vcom application on seed germination (A-B), shoot length (C), shoot diameter (D) and root length (E) of the seedlings of *W*. *somnifera*. DW: distilled water; KN: KNO_3_, Vcom: Vermicompost; Vcom-L: Vermicompost leachate; Vcom-T: Vermicompost tea. Letters (a, b, c, d) marked statistical significance (HSD, P≤0.05) within various pre-soaking treatments (DW, KN, Vcom-L and Vcom-T); whereas letters (p, q, r, s) represented statistical significance (HSD, P≤0.05) between various concentrations of Vcom (0, 20, 40, 60, 80 and 100%) performed by ANOVA test.

*W*. *somnifera* seeds, before sowing, were pre-soaked in DW, KN, Vcom-L or Vcom-T for either 1 h or 12 h. The treated seeds were then sowed in soil containing different proportions (%) of Vcom. As shown in Fig 1A and 1B, we found that irrespective of various pre-soaking treatments, 12 h treatment group performed better than 1 h group in germination efficacy. Furthermore, seeds pre-soaked in Vcom-L and grown in control soil showed high germination (38.75±0.75%) as compared to the ones pre-soaked in Vcom-T, KN or DW. Various combinations further revealed that the germination was improved (p≤0.001) on supplementing the soil with Vcom (Fig 1B). Maximum germination (81.25±0.63% and 72.3% higher than control) was recorded for seeds pre-soaked in DW for 12 h and grown in 80% Vcom. This value was significantly higher (p≤0.001) than all other pre-soaking groups (KN, Vcom-L and Vcom-T). Furthermore, to analyse the promotive effect of Vcom and Vcom-L, we performed element analysis. It was observed that sand: soil possessed C-1.14%, N- 0.39%, P- 0.03 g/kg and K-3.63 g/kg; Vcom contained C-12.47%, N- 0.55%, P- 0.16 g/kg and K- 6.85 g/kg, and Vcom-L comprised C- 0.03%, N- 0.37%, P-0.24 mg/L and K- 631.2 mg/L.

**Fig 2:**
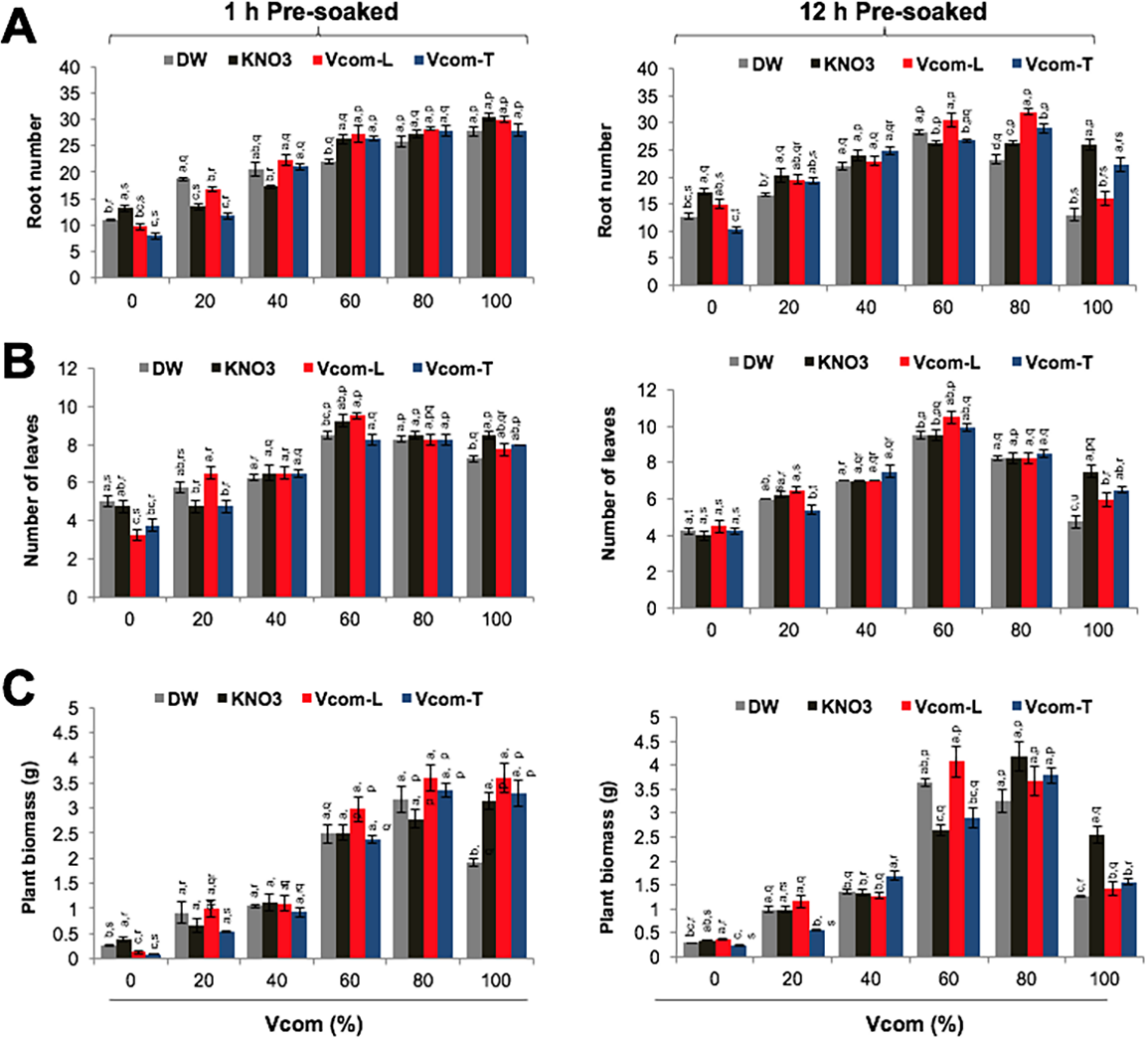
Effect of pre-soaking on *W*. *somnifera* vegetation and biomass. Effect of pre-soaking and Vcom application on root number (A), number of leaves (B) and seedling biomass (C). Letters (a, b, c, d) marked statistical significance (HSD, P≤0.05) within various pre-soaking treatments; whereas letters (p, q, r, s) represented statistical significance (HSD, P≤0.05) between various concentrations (%) of Vcom performed by ANOVA test.

Forty five days-old seedlings were examined for morphological parameters. As shown in Figs 1 and 2, 12 h pre-soaking for all the 4 groups resulted in better growth than 1 h pre-soaking group. We found that the seeds pre-soaked in DW (1 h) and sowed in control (0% Vcom) resulted in shortest shoot length (0.88±0.08 cm) and this steadily increased (p≤0.001) for seeds sowed in Vcom-supplemented soil (Fig 1C). Maximum shoot length (9.53±0.55 cm) was observed with 80% Vcom for Vcom-L soaked seeds from 1 h group. Comparison of shoot diameter (cm) in control revealed that the pre-soaking in Vcom-T resulted in thicker shoots (0.33±0.01) (Fig 1D). Furthermore, a combination of Vcom-T pre-soaking (12 h) and 80% Vcom caused maximum increase in diameter (0.47±0.01), which differed significantly (p<0.001) from DW and KN pre-soaking (Fig 1D).

Examination of root length (cm) in control showed that the longest roots (8.55±0.31) were present in Vcom-T pre-soaked group (Fig 1E). Vcom supplementation resulted in a further increase in root length. Maximum length (9.13±0.49) was obtained in Vcom-T pre-soaked and 40% Vcom sowed group. Root number/seedling showed highest increase with KN (17.25±0.62) pre-soaking, in control (0% Vcom). A significant (p≤0.001) increase was observed after Vcom supplementation. Maximum increase was recorded in Vcom-L pre-soaked, 80% Vcom plants (32.0±0.59), which was 53% more than control (Fig 2A). Leaf number/seedling in control was highest in 1 h DW pre-soaking group (5.0±0.27) (Fig 2B). Vcom supplementation resulted in an increase in leaf number and was more pronounced in 12 h pre-soaking group. Maximum increase (57% more than control) was recorded in Vcom-L pre-soaked (12 h) and 60% Vcom (10.5±0.33) cultivated plants (Fig 2B). In control, total biomass (g/seedling) was maximum in 12 h Vcom-L (0.37±0.02) pre-soaked group (Fig 2C). A significant increase (92% more than control; p≤0.001) was noticed with Vcom supplementation. Highest biomass was recorded for 12 h KN pre-soaked, 80% Vcom (4.19±0.31) plants (Fig 2C).

### Effect of pre-soaking, Vcom and Vcom-L on plant growth and yield

**Fig 3:**
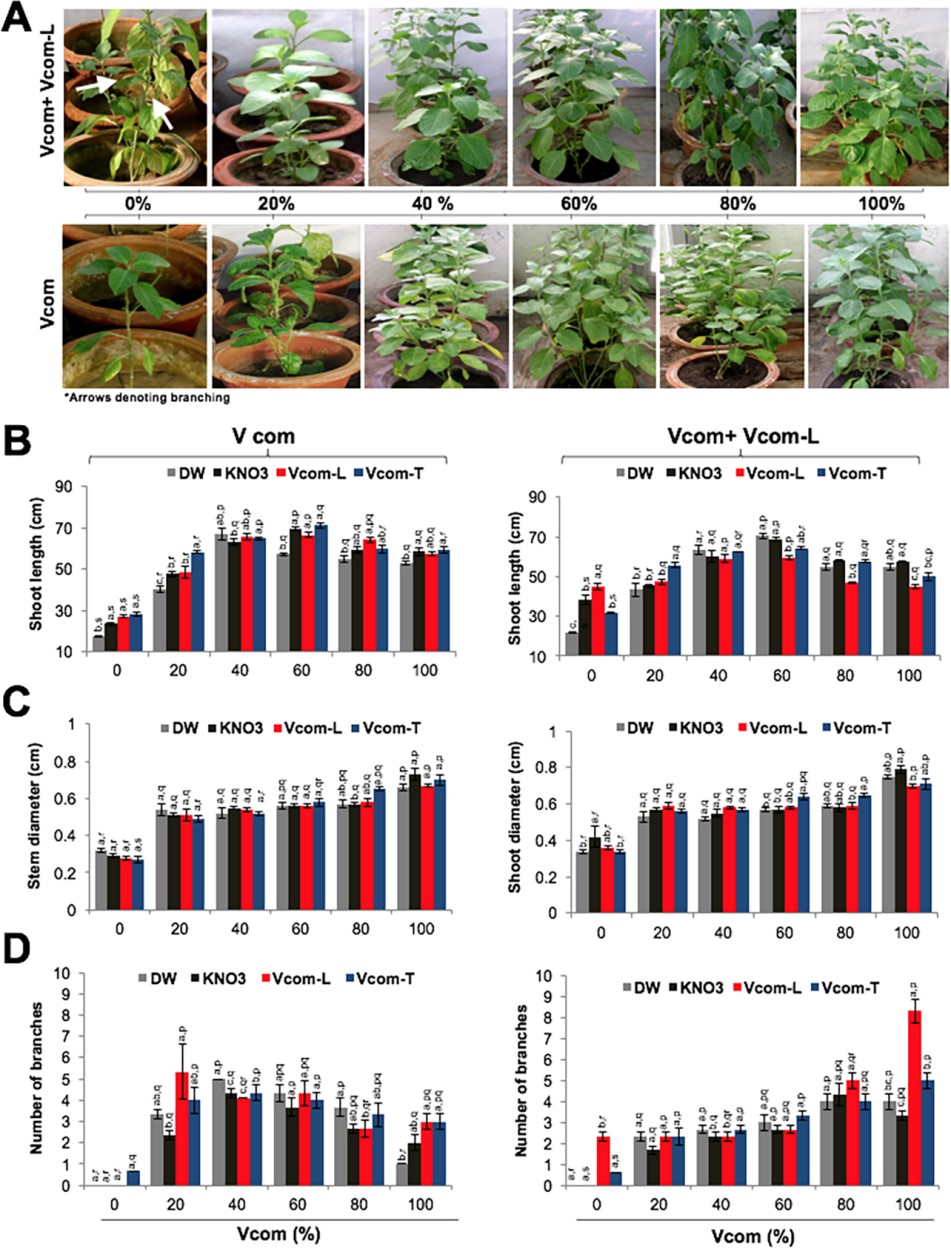
Effect of Vcom and Vcom+Vcom-L on *W*. *somnifera* growth. Figure showing effect Vcom and Vcom+Vcom-L application on plant growth (A), shoot length (B), shoot diameter (C), and number of branches (D) of *W*. *somnifera*. Letters (a, b, c, d) marked statistical significance (HSD, P≤0.05) within various pre-soaking treatments; whereas letters (p, q, r, s) represented statistical significance (HSD, P≤0.05) between various concentrations (%) of Vcom performed by ANOVA test.

Investigations on the effect of pre-soaking, Vcom and Vcom-L supplementation on the morphological parameters of sixty days old plants showed that binary combination of Vcom and Vcom-L resulted in better growth of plants (p<0.001) (Fig 3A). A maximum increase in shoot length (60% over control) was observed for Vcom-T pre-soaked (71.2±1.21cm) 60% Vcom grown plants in the control group (Fig 3B). However, in the Vcom-L group it was maximum for DW pre-soaked plants. For shoot diameter (cm) DW pre-soaked plants showed highest value in 0% Vcom group (Fig 3C). An increase (p≤0.001) was observed with Vcom supplementation regardless of pre-soaking treatment (Fig 3C). Vcom-L treatment caused highest increase (0.79±0.02, 26% over control) in KN pre-soaked, 100% Vcom grown plants. DW, Vcom-L and Vcom-T pre-soaked seeds sowed in 100% Vcom gave similar, although slightly less pronounced results (Fig 3C).

Number of branches/plants showed a strong improvement up to 60% Vcom irrespective of various pre-soaking treatments (Fig 3D). Plants in 0% Vcom did not show branching while Vcom-T induced branching even in this group. Vcom supplementation caused increase in branching in a concentration dependent manner (maximum with 60%). Vcom-L application caused a further increase in branching/plant (Fig 3D). Maximum number of branches reaching a value of 72% more than control (p≤0.001) was recorded for Vcom-L pre-soaked, 100% Vcom+Vcom-L plants (8.33±0.56). This value was significantly higher than DW, KN and Vcom-T pre-soaked groups (Fig 3D).

**Fig 4:**
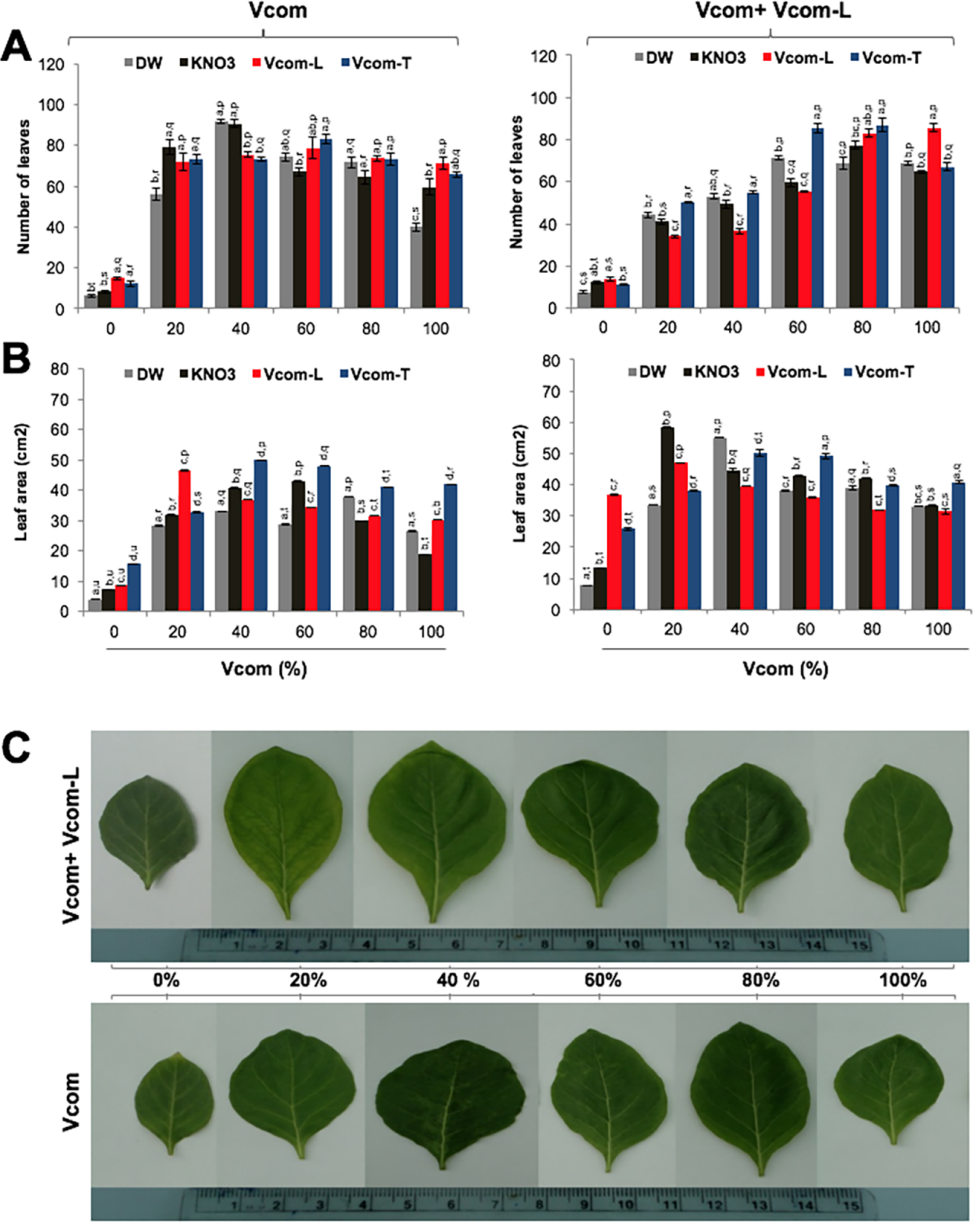
Effect of Vcom and Vcom+Vcom-L on *W*. *somnifera* vegetative development. Effect of pre-soaking, Vcom and Vcom-L on number of leaves (A), leaf area (B) of *W*. *somnifera*. Whereas, size of the leaves of *W*. *somnifera* grown with Vcom and Vcom+Vcom-L is shown below (C). Letters (a, b, c, d) marked statistical significance (HSD, P≤0.05) within various pre-soaking treatments; whereas letters (p, q, r, s) represented statistical significance (HSD, P≤0.05) between various concentrations (%) of Vcom performed by ANOVA test.

In control (0% Vcom), leaf number/plant was highest in Vcom-L (15.0±0.63) pre-soaked group (Fig 4A). In comparison to control, Vcom supplementation caused a significant (p≤0.001) increase in leaf number regardless of pre-soaking treatments (Fig 4A). Highest increase (93% over control) was recorded for DW pre-soaked (91.67±1.12) and 40% Vcom plants. This value was significantly different from Vcom-L and Vcom-T groups. Strong effect of Vcom-L treatment was evident till 100% Vcom, where the leaf number for all the pre-soakings was significantly more than control plants (p<0.001) (Fig 4A).

In 0% Vcom plants, leaf area (cm^2^) was highest in Vcom-T (15.74±0.05) pre-soaked group (Fig 4B). Vcom-L treatment caused 2–3 fold increase in the leaf area (7.83±0.03–36.82±0.15). Vcom in the potting mixtures caused a significant increase in leaf area (p≤0.001). Binary combination of Vcom (up to 40%) and Vcom-L caused more enhancements in leaf area irrespective of pre-soaking treatments (Fig 4B and 4C). Largest increase of 77% over control was recorded for KN pre-soaked, 20% Vcom+Vcom-L plants (58.28±0.24). This value was significantly higher than the values obtained for DW, Vcom-L and Vcom-T pre-soaked plants. Higher proportions (60–100%) of Vcom caused slight reduction in leaf area (Fig 4C).

**Fig 5:**
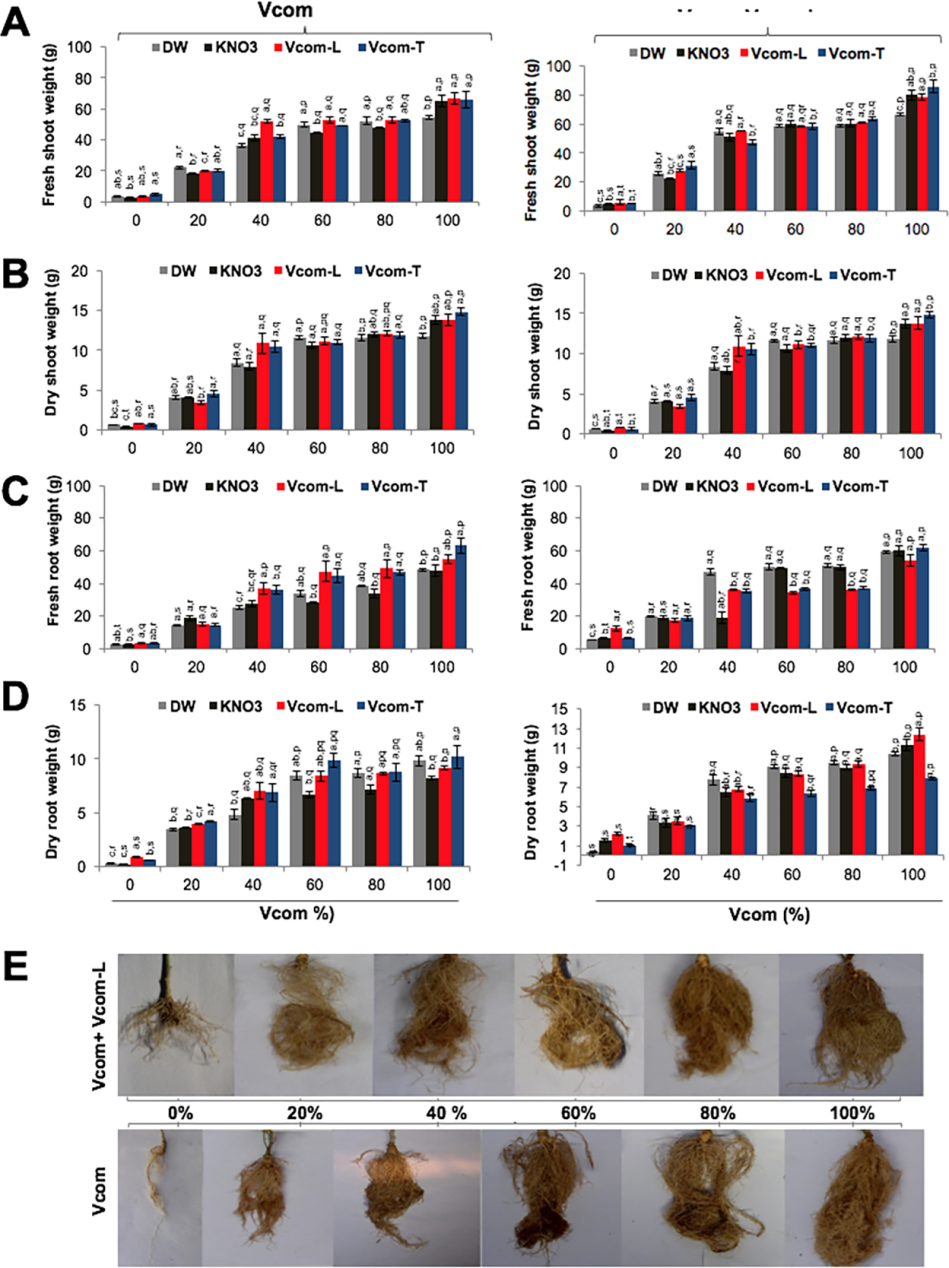
Effect of Vcom and Vcom+Vcom-L on *W*. *somnifera* vegetative extension. Effect of pre-soaking, Vcom and Vcom-L on fresh and dry weight of shoot (A, B) and root (C, D) of *W*. *somnifera*. Whereas, size of the roots of *W*. *somnifera* grown with Vcom and Vcom+Vcom-L is shown below (E). Letters (a, b, c, d) marked statistical significance (HSD, P≤0.05) within various pre-soaking treatments; whereas letters (p, q, r, s) represented statistical significance (HSD, P≤0.05) between various concentrations (%) of Vcom performed by ANOVA test.

Fresh and dry weight (shoot and root/plant) of seventy five days old plants as represented in Fig 5A–5D, showed that in 0% Vcom group, Vcom-T pre-soaking was better (4.86±0.59) for fresh shoot weight (g). With Vcom supplementation, fresh shoot weight showed a dose dependent increase (p≤0.001) irrespective of various pre-soaking treatments. Vcom-L treatment of plants resulted in further improvement in fresh shoot weight and maximum increase (94% over control) was observed for Vcom-T (85.71±4.02) pre-soaked 100% Vcom grown plants (Fig 5A). This value was significantly higher than DW and Vcom-L pre-soaked groups. Dry shoot weight (g) was highest in Vcom-L (0.83±0.05) pre-soaked, 0% Vcom grown plants (Fig 5A). On supplementing the potting mixture with Vcom, a significant (p≤0.001) improvement in dry shoot weight was observed with a maximum value at 100% Vcom (Fig 5B). Subsequent treatment with Vcom-L resulted in higher increase in dry shoot weight in all the groups. Highest increase in the dry shoot weight was 91% more than control in KN pre-soaked (20.08±0.51) 100% Vcom+Vcom-L plants (Fig 5B). This value was significantly more than DW and Vcom-T plants.

Fresh root weight (g) was highest in Vcom-L (3.61±0.51) pre-soaked 0% Vcom-grown plants that further increased to 12.64±1.54 with Vcom-L supplementation (Fig 5C–5E). A significant improvement was observed with Vcom supplementation (p≤0.001). Maximum increase (63.17±4.62; 95% over control) was recorded for Vcom-T pre-soaked plants grown in 100% Vcom (Fig 5C). Dry root weight (g) was also maximum in Vcom-L pre-soaked (0.92±0.09) 0% Vcom group. A significant increase (p≤0.001) with Vcom increased further with Vcom-L application (Fig 5D). Maximum increase in Vcom-L treated plants (~82% more than control) was recorded for Vcom-L pre-soaked (12.38±0.66) plants grown in 100% Vcom.

**Fig 6:**
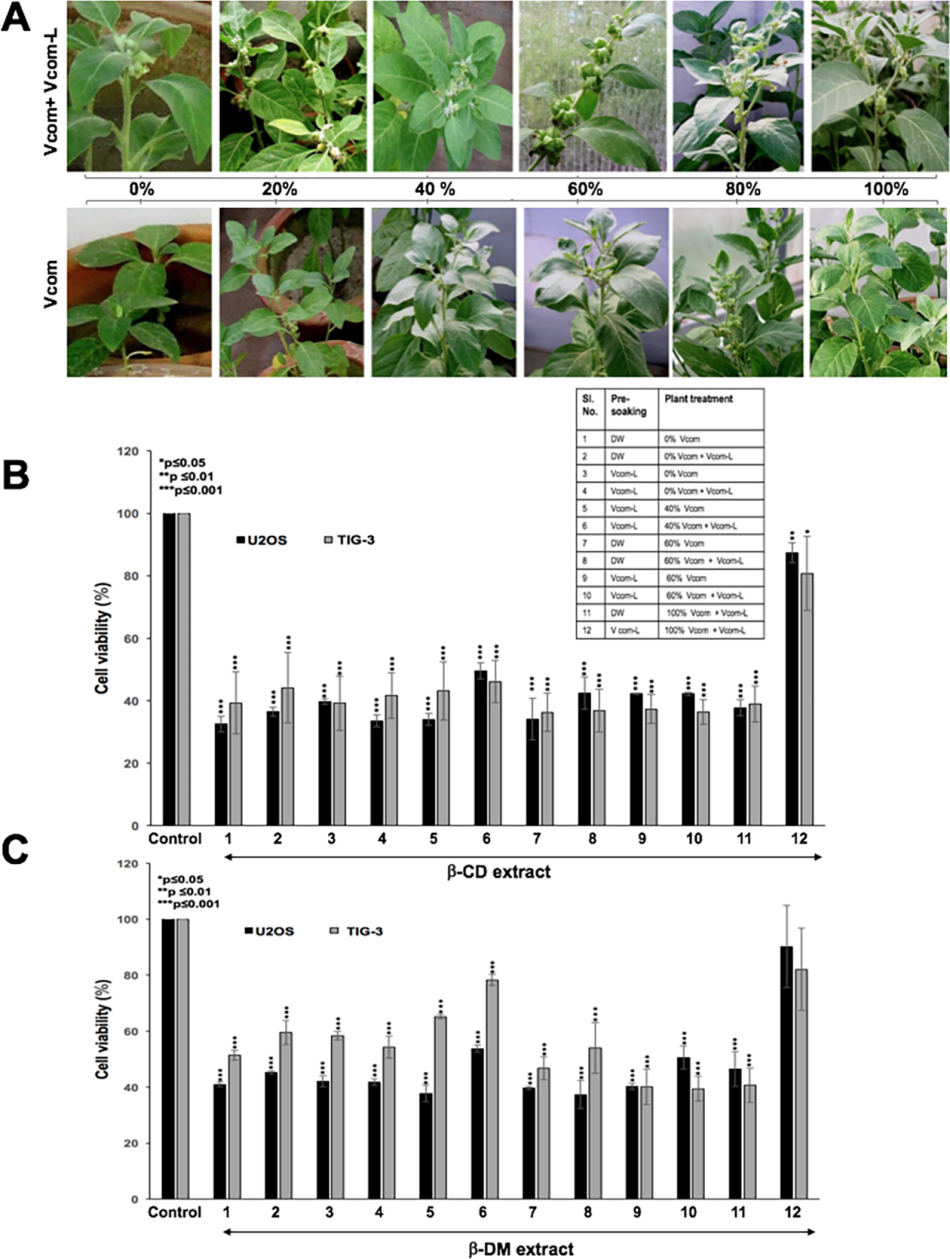
Effect of Vcom and Vcom+Vcom-L on *W*. *somnifera* fruiting and analysis of cell cytotoxicity treated with leave extracts. Effect of Vcom and Vcom+Vcom-Lon flowering and fruiting of *W*. *somnifera* (A). Cytotoxicity of leaf extracts (β-cyclodextrin assisted water extract, β-CD, and its residual fraction dissolved in DMSO, β-DM) in human cancer and normal cells is shown (B-C). Whereas the extracts from plants pre-soaked with Vcom-L and treated with Vcom (up to 60%) showed cytotoxicity, the extracts from plants treated with 100% Vcom were inactive. Furthermore, β-CD leaf extracts showed somewhat equal cytotoxicity to both human cancer and normal cells; β-DM extracts from plants treated with 40% Vcom showed highest selectivity to human cancer cells (B-C).

The performance of *W*. *somnifera* under the influence of Vcom and Vcom-L was also assessed with respect to onset of flowering and fruiting (Fig 6A). It was found that the potting mixtures supplemented with 60% Vcom showed earliest flowering and fruiting (45 and 57 days, respectively, from the day of transplantation) (Fig 6A). At higher proportions of Vcom (80 and 100%), flowering was observed after 48–55 days and fruiting after 65–70 days. However, in 20% and 40% Vcom, although flowering occurred after 46 days of transplantation, flower showed tendency to shed after some time hampering the process of fruit formation. Vcom-L supplementation initiated flowering even in 0% Vcom plants after 75 days of transplantation and considerably reduced the time of flowering (45–50 days) and fruiting (50–67 days) in 80 and 100% Vcom, respectively (Fig 6A).

### Effect of seed pre-soaking, Vcom and Vcom L on the quantity of withanolides and analysis of cell cytotoxicity with leave extracts

HPLC analysis showed (Table 1 and S2–S5 Figs) that 40% Vcom substitution resulted in higher levels of Withanolides (Wi-A, Wil-A and Wi-N) that increased further with Vcom L application. Highest content of Wi-A (4.89±0.14 mg/g; ~80% higher than the control), Wil-A (0.06 ±0.004 mg/g; ~50% over control) and Wi-N (5.13±0.13 mg/g; ~50% over control) was recorded for DW pre-soaked plants. Although a large variety of secondary metabolites including Withanolides have been detected in *Withania somnifera* extracts, most bioactivities have been ascribed to bioactive Withanolides, Wi-A, Wi-N and Wil-A. We focused on Wi-A and Wi-N that have been reported to possess considerable anticancer activity. Whereas Wi-A has been shown to cause strong cytotoxicity to cancer and normal cells, Wi-N was mild but safe for normal cells [5–7, 12, 13, 25]. We have earlier reported the protocols to obtain Withanolides in water extract using alpha, beta and gamma cyclodextrins. Beta-cyclodextrin (β-CD) extracts showed higher concentration of both Wi-N and Wi-A [12]. Wi-N, being hydrophobic, remained insoluble and was found in the pellet. Twelve samples of leaves raised under different conditions (Fig 6B) were extracted using β-cyclodextrin. The withanolide (Wi-N) that remained in the residual mixture/pellet was further extracted with DMSO (called β-DM). Both β-CD and β-DM extracts were examined for their anticancer bioactives by established viability assays. In agreement with our earlier study [12], we found that the 0.05% β-CD extracts were cytotoxic to human cancer cells suggesting that the leaves contained the bioactive withanolides (Wi-N and Wi-A). Furthermore, they also showed equivalent cytotoxicity to human normal cells (Fig 6B). The latter was attributed to the presence of Wi-A in these extracts. The β-DM extracts that possessed high ratio of Wi-N showed somewhat higher toxicity to human cancer cells as compared to the normal cells (Fig 6C). These data suggested that the plants raised in the present study are suitable for their use in anticancer and other benefits that have been ascribed to active Withanolides.

**Table 1:** Effect of pre-soaking, Vcom and Vcom-L application on the content of Withaferin A, Withanolide A and Withanone in leaves of *W*. *somnifera*.

Vcom conc. (%)	Seed pre-soaking			Withanolides (mg/g dry wt)			
		Withaferin A Vcom Vcom+Vcom-L		Withanolide A Vcom Vcom+Vcom-L		Withanone Vcom Vcom+Vcom-L	
0	DW	1.7 ±0.02^a,p^	3.9±0.09^a,p^	0.019±0.002^a,p^	0.03±0.002^a,p^	2.3±0.04^a,p^	4.7±0.04^a,p^ 3.7±0.24^b,p^ 4.6±0.19^a,p^
	Vcom-L	1.6±0.01^b,p^	3.1±0.20^b,p^	0.026±0.002^a,p^	0.03±0.001^a,p^	2.4±0.08^a,p^	
	Vcom-T	2.6±0.03^c,p^	4.0±0.03^a,p^	0.029±0.002^a,p^	0.038±0.003^c,p^	2.7±0.13^b,p^	
40	DW	4.2±0.02^a,q^	4.9±0.14^a,q^	0.039±0.01^a,q^	0.06±0.004^a,q^	3.8±0.05^a,q^	5.1±0.13^a,q^ 2.8±0.02^b,q^ 4.7±0.03^c,p^
	Vcom-L	3.6±0.03^b,q^	2.4±0.02^b,q^	0.04±0.003^a,q^	0.03±0.001^b,p^	3.9±0.03^a,q^	
	Vcom-T	4.4±0.06^c,q^	4.6±0.03^c,q^	0.048±0.003^a,q^	0.045±0.002^c,q^	4.6±0.05^b.q^	
60	DW	2.3±0.02^a,r^	3.0±0.03^a,r^	0.028±0.001^a,p^	0.031±0.001^a,p^	3.4±0.02^a,r^	3.9±0.02^a,r^ 2.3±0.03^b,r^ 3.7±0.02^a,q^
	Vcom-L Vcom-T	3.3±0.02^b,r^ 3.7±0.11^c,r^	2.2±0.01^b,q^ 2.9±0.01^a,r^	0.029±0.002^a,p^ 0.042±0.002^b,q^	0.036±0.001^a,q^ 0.037±0.002^a,p^	3.2±0.02^b,r^ 3.9±0.02^c,r^	

Values are represented as mean ± SE (n = 3). Along the column, letters—a, b and c representing statistical significance (HSD, P≤0.05) within various pre-soaking (DW, Vcom-L and Vcom-T) treatments; whereas letters—p, q and r represent statistical significance among various Vcom concentrations (0, 20, 40, 60, 80 and 100%) as calculated by ANOVA.

## Discussion

The present study investigated the potential role of Vcom and its liquid preparations (Vcom-L and Vcom-T) on seed germination, growth and yield of *W*. *somnifera*. Germination and seedling emergence are two critical phases for establishment of a crop, which along with an internal regulation are affected by external factors like temperature, moisture, presence or absence of hormonal stimulators and inhibitors. Pre-soaking of seeds has been known to stimulate metabolic activities, soften hard seed coat, leaching of chemical inhibitors of germination and ensure rapid and uniform germination [14–16]. In the present study, pre-soaking for 12 h appeared to remarkably influence germination and growth of *W*. *somnifera* seedlings that further improved with supplementation of Vcom to growth mixtures. These results are in conformity with other studies that documented stimulatory action of pre-soaking [32] and Vcom application on seed germination and growth of seedlings of peppers and petunias [33, 34]. Positive influence of Vcom-L and Vcom-T pre-soaking on the morphology of seedlings in the present work is supported by earlier findings in pea and tomato seedlings [27,35]. Vcom enriches the soil with polysaccharides that act as cementing substance to enhance aggregate stability for better aeration, water retention, drainage, aerobic condition of soil and nutrient availability [36] and directly stimulates plant growth. Additionally the humic substances present in Vcom activate enzymes for nitrate metabolism, organogenesis and plant growth [37]. Furthermore, our study showed that the different morphological parameters of seedlings responded differentially to different proportions of Vcom. Maximum increase for most of the parameters was observed upto 80% Vcom, 100% Vcom however did not cause further stimulation of germination and seedling growth, rather all the parameters showed a decrease in this mixture. It is consistent with other reports that have demonstrated that growth and yield of plants decreased when more than 60% Vcom was incorporated into the growth mixtures [38–40]. These studies correlated decrease in germination and growth of seedlings to high concentration of salts and phenolic substances at higher concentrations of Vcom.

Vcom-L was used as an additional supplement along with Vcom to enhance the growth of *W*. *somnifera* at later stages of its development. It has been suggested that Vcom-L has potential to act as a growth stimulator when used as a soil drench or foliar spray [41]. Interestingly, our results also indicated an additional enhancement in plant growth under the combined effect of Vcom and Vcom-L. Regardless of pre-soaking, growth and yield of this plant showed evident increase with increasing percent (up to 60%) content of Vcom in the potting mixture. For instance, 40–60% Vcom was better for shoot length and 20–60% Vcom was beneficial in improving leaf number and leaf area. Moreover, maximum increase in leaf number (14-fold more than control) and leaf area (4-fold more than control) in our study under the influence of the binary combination of Vcom+Vcom-L indicated more biomass accumulation, very important to bridge the gap between demand and supply of *W*. *somnifera*. These results are in line with a recent finding on three medicinal plants i.e. *Eucomisautumnalis*, *Tulbaghialudwigiana and Tulbaghiaviolacea*, where an increase in leaf number and leaf size has been reported with the application of Vcom-L [42]. Similarly, shoot weight (fresh and dry) is an indicator of total biomass allocated to the aerial parts of a plant, therefore enhancement of shoot diameter and number of branches in the present work can be linked to a concomitant highest increase in plant biomass in 100% Vcom. Furthermore, growth of a plant could be assigned to its efficient root system. A steady increase in root growth was observed with percentage of Vcom in our study. Similar results have been reported for tomato and olive plants with incorporation of Vcom [43] and humic acids extracted from Vcom [44]. We also observed a significant enhancement in root growth in the form of dry weight after Vcom-L application. Enhancement of growth parameters of *W*. *somnifera* with this binary combination can be attributed to increase in phytoassimilable nutrients, porosity and moisture retention with the concentration of Vcom in the potting mixtures and higher content of soluble/exchangeable nutrients in Vcom-L. The humic substances present in Vcom have been reported to activate ion channels (H^+^-ATPase) present on the root plasma membrane and stimulate differentiation of sites of lateral root emergence [45, 46]. Such an effect of humic substances could be correlated to enhancement of nutrient uptake by the plant [47, 48]. Hence, improvement in the root system under the combined effect of Vcom and Vcom-L may have enabled the plant to optimally exploit the available resources for its growth and development.

Another possible reason for the observed enhancement of growth and yield of *W*. *somnifera* may be the occurrence of some bio-stimulants and plant growth regulators of microbial origin in Vcom and its liquid preparations. These might have promoted a series of signalling events that affected plant metabolism like cell respiration, photosynthesis, protein synthesis and other enzymatic reactions as suggested in other studies [49–51]. Recently, presence of cytokinins, auxins, abscisic acid, gibberellins, brassinosteroids and phenolic compounds has been confirmed in garden waste derived Vcom-L [52]. Moreover, hormonal nature of Vcom-derived humic acids has been reported due to similarity in their action to plant growth regulators such as IAA [53]. This suggests the possibility that these hormones may be attached to humic acid molecules and thereby directly influences development of a plant when a growth mixture is supplemented with Vcom and Vcom-L [54].

Of note, we also found that 60% Vcom cultivated plants showed earlier onset of flowering and fruiting while 80% and 100% Vcom resulted in a slight delay (~1 week) in onset of flowering as well as fruiting (Fig 3). The data is supported by the findings of Sangwan et al. [55] who reported reduction in the time of flowering (by 90 days over control) of Marigold at 30% and 40%, with maximum number of flowers at 30% vermicompost. The binary combination of Vcom and Vcom-L, however, induced flowering and fruiting in 80% and 100% Vcom at almost the same time to that of 60% Vcom plants in our study. Molecular mechanism(s) of such an effect of Vcom and Vcom-L on the flowering and fruiting warrants further studies.

Improvement in growth of *W*. *somnifera*, in the present study, is directly related to the significant rise in the contents of Withanolides under the combined effect of Vcom+Vcom-L. This was further confirmed through cytotoxicity assays using human cancer and normal cells. As expected, β-cyclodextrin assisted extracts, β-CD and β-DM [12] showed cytotoxicity in the range of 0.05~0.1% in culture medium. Furthermore, leaf extracts from plants raised under conditions, such as pre-soaking in Vcom-L and treated with combined Vcom (up to 60%) + Vcom-L showed good anticancer activity. On the other hand, leaf extracts from plants treated with 100% Vcom + Vcom-L showed low activity suggesting that they did not possess active Withanolides (Fig 6B and 6C). The result was endorsed by data from both ®-CD and β-DM extracts. Furthermore, β-CD extracts showed equivalent cytotoxicity to human cancer and normal cells (Fig 6B), the β-DM extracts were more cytotoxic to cancer cells (Fig 6C). The data was in agreement with the expected higher content of Wi-A in β-CD extract and Wi-N in β-DM extract [5,12]. Since experimental evidence of various activities in the leaves of *W*. *somnifera* and active Withanolides (Wi-A and Wi-N) has been rapidly accumulating, the current results describing the use of Vcom for increasing their biomass and bioactive content are important to enhance its medicinal value.

## Conclusions

Pre-soaking in Vcom-L and Vcom-T for 12 h is recommended as an alternative to the chemical treatments to improve seed germination and seedling establishment of *W*. *somnifera*. Addition of 60% Vcom in the soil boosted germination, growth and yield of the plant, and also shortened the time to obtain reproductively mature plants. Vcom-L application effectively enhanced the growth, yield and bioactive compounds in the plants when used in combination with Vcom. Hence the data suggest that the use of Vcom and its by-products (Vcom-L and Vcom-T) to cultivate *W*. *somnifera* will effectively enhance its potential use as a functional food and phytopharmaceutical agent.

## Supporting information

S1 Fig Schematic diagrams showing components and organization of Vcom-L collection unit. (TIF)

S2 Fig HPLC profiles showing the standards of HPLC analysis. (TIF)

S3 Fig HPLC profiles showing quantity of withanolides (Wi-A, Wil-A and Wi-N) in leaves of *W*. *somnifera* from DW, Vcom-L and Vcom-T pre-soakings at 0% Vcom, with (Vcom+Vcom-L group) and without (Vcom group) Vcom-L application. (TIF)

S4 Fig HPLC profiles showing quantity of withanolides (Wi-A, Wil-A and Wi-N) in leaves of *W*. *somnifera* from DW, Vcom-L and Vcom-T pre-soakings at 40% Vcom, with (Vcom+Vcom-L group) and without (Vcom group) Vcom-L application. (TIF)

S5 Fig HPLC profiles showing quantity of withanolides (Wi-A, Wil-A and Wi-N) in leaves of *W*. *somnifera* from DW, Vcom-L and Vcom-T pre-soakings at 60% Vcom, with (Vcom+Vcom-L group) and without (Vcom group) Vcom-L application. (TIF)
