# Supplementary figures and images for: Organic cultivation of Ashwagandha with improved biomass and high content of active Withanolides: Use of Vermicompost
Source: PLoS One. 2018 Apr 16;13(4):e0194314. doi: 10.1371/journal.pone.0194314 (PMC5901777; doi:10.1371/journal.pone.0194314)

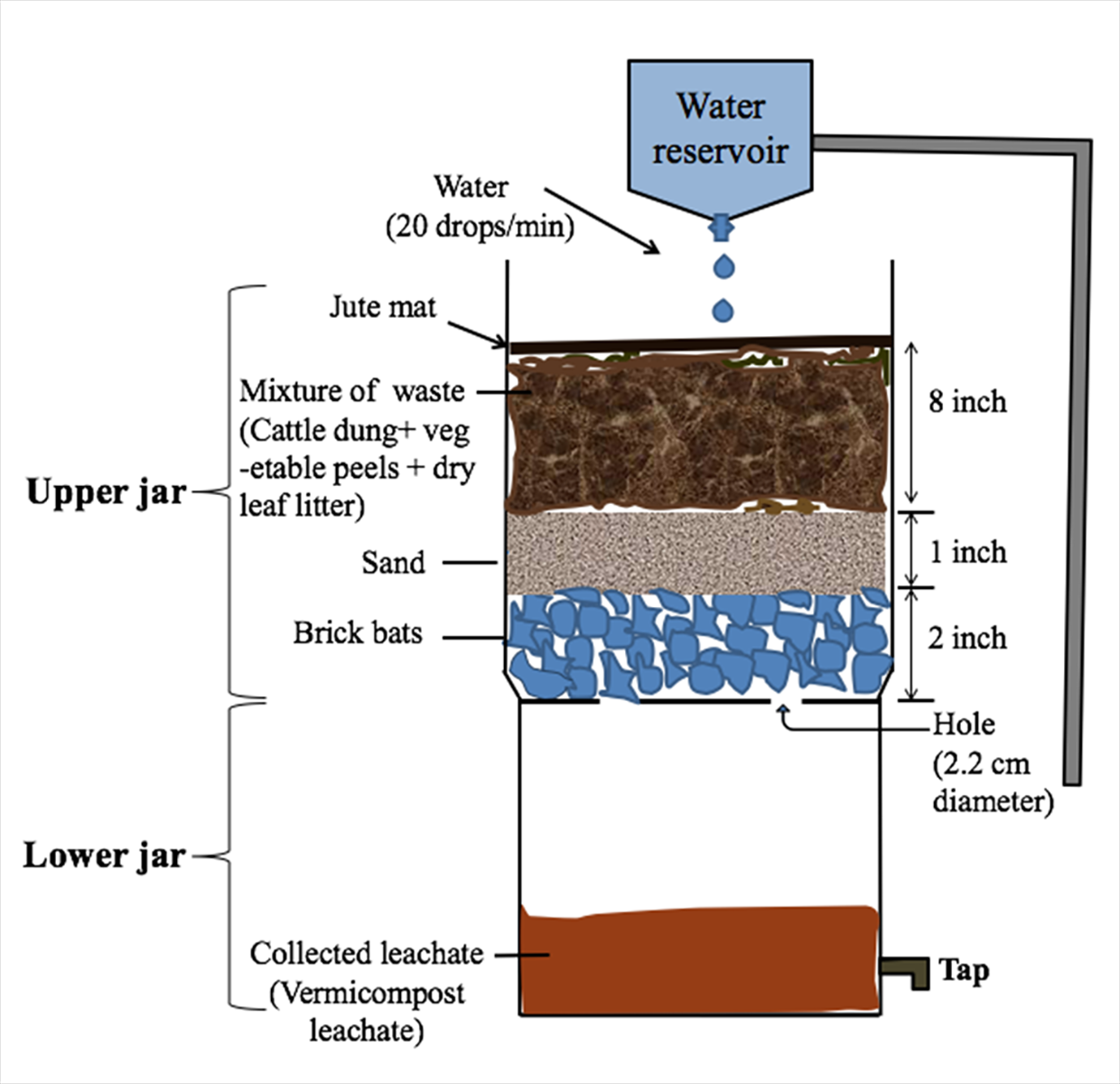

Supplement: S1 Fig — (TIF) [file pone.0194314.s001.tif]

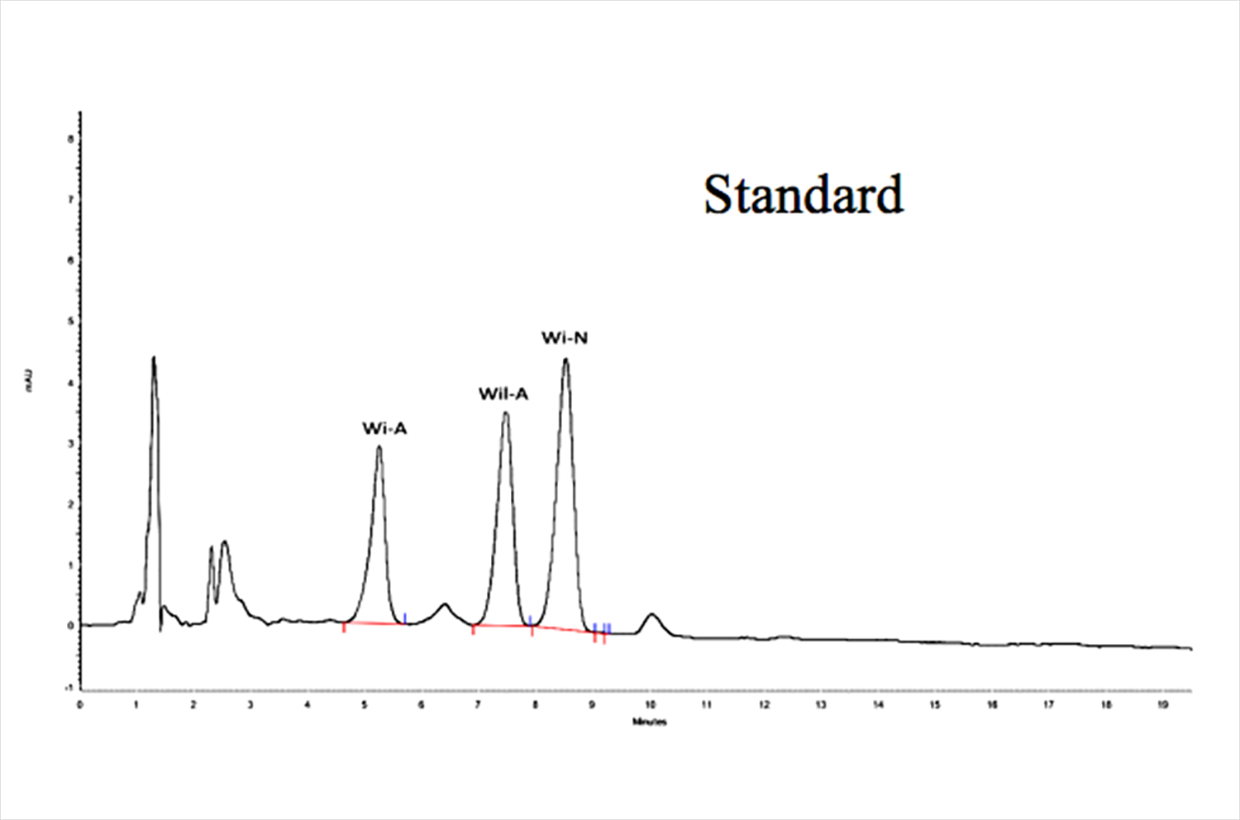

Supplement: S2 Fig — (TIF) [file pone.0194314.s002.tif]

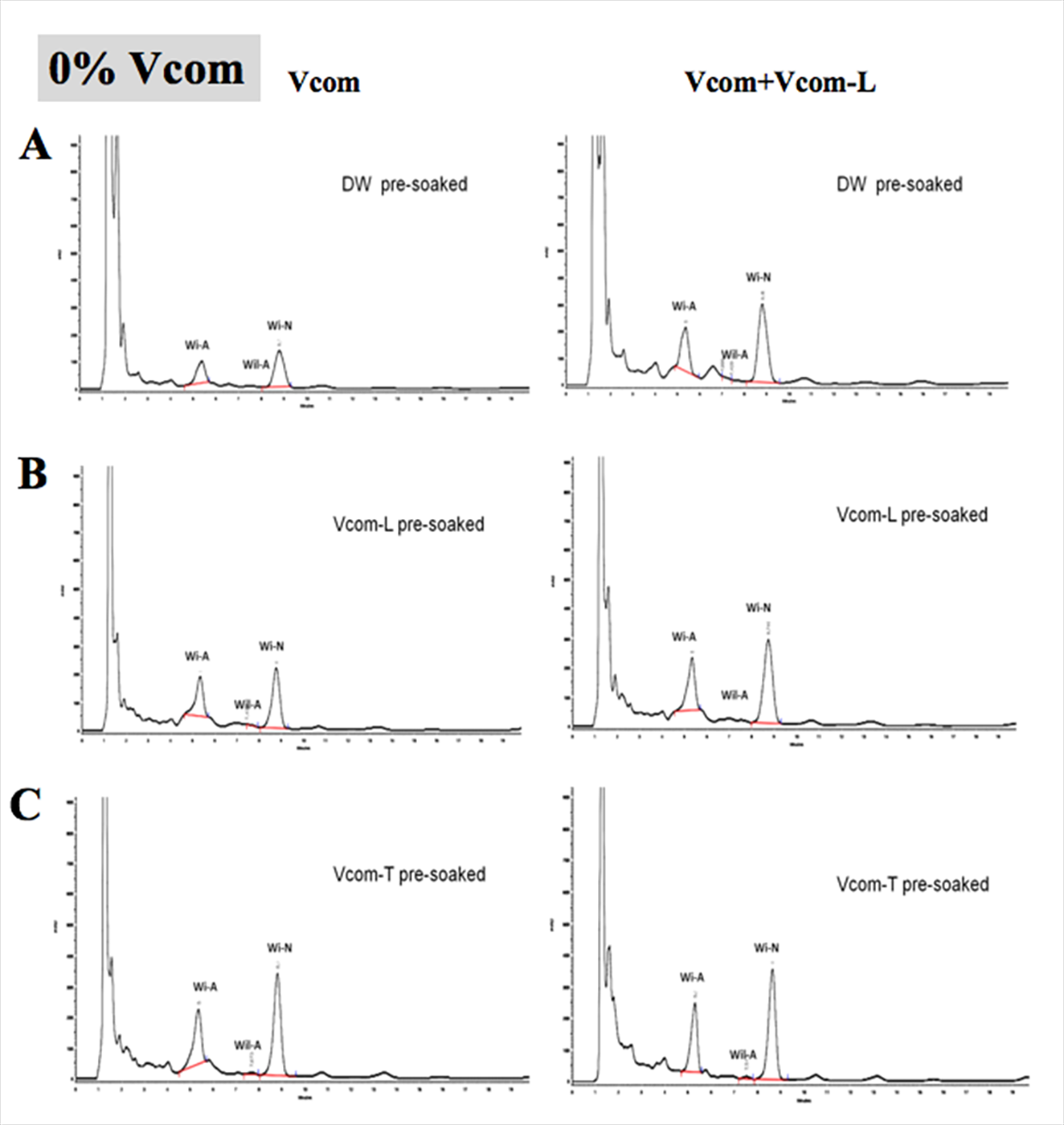

Supplement: S3 Fig — (TIF) [file pone.0194314.s003.tif]

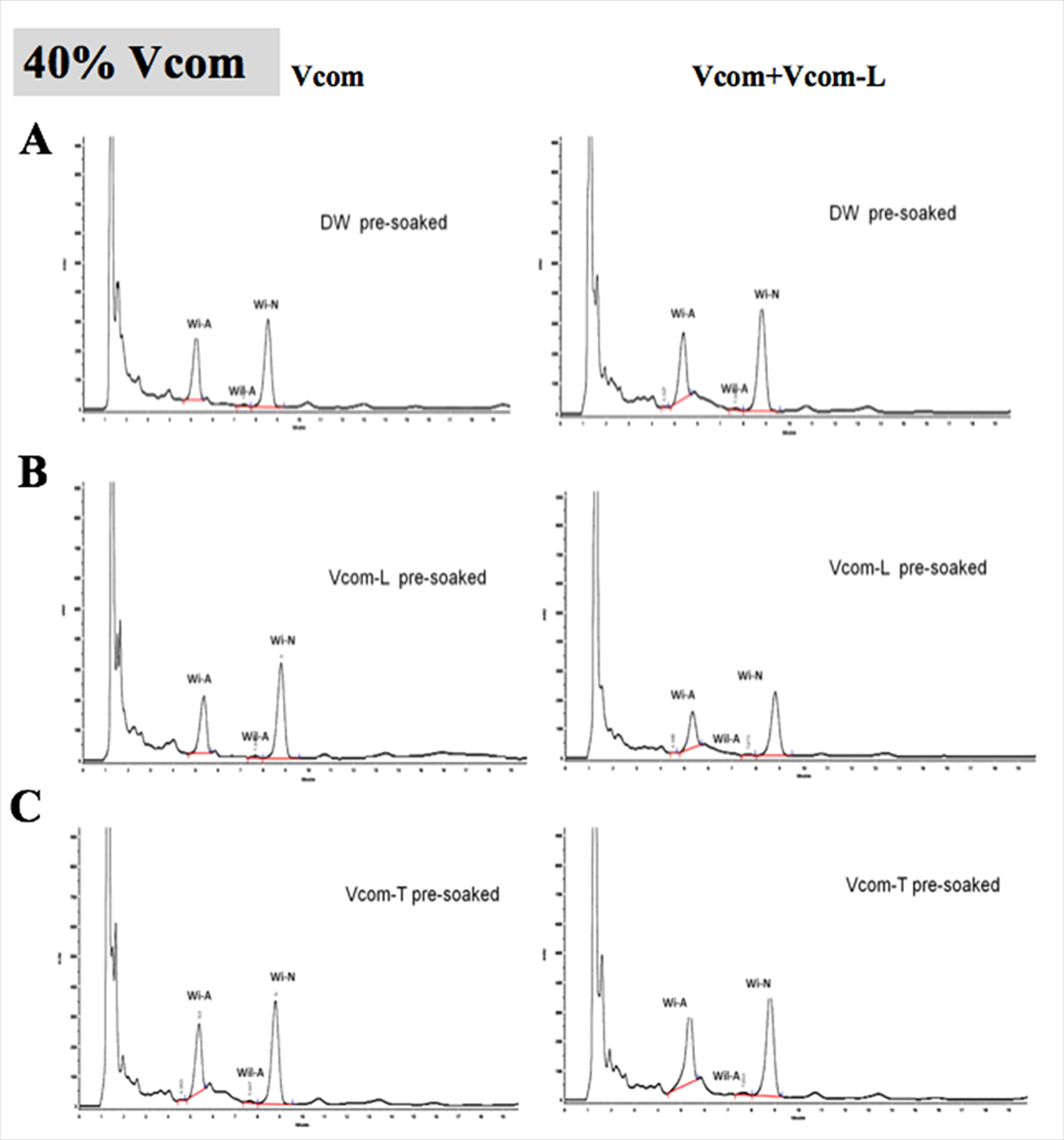

Supplement: S4 Fig — (TIF) [file pone.0194314.s004.tif]

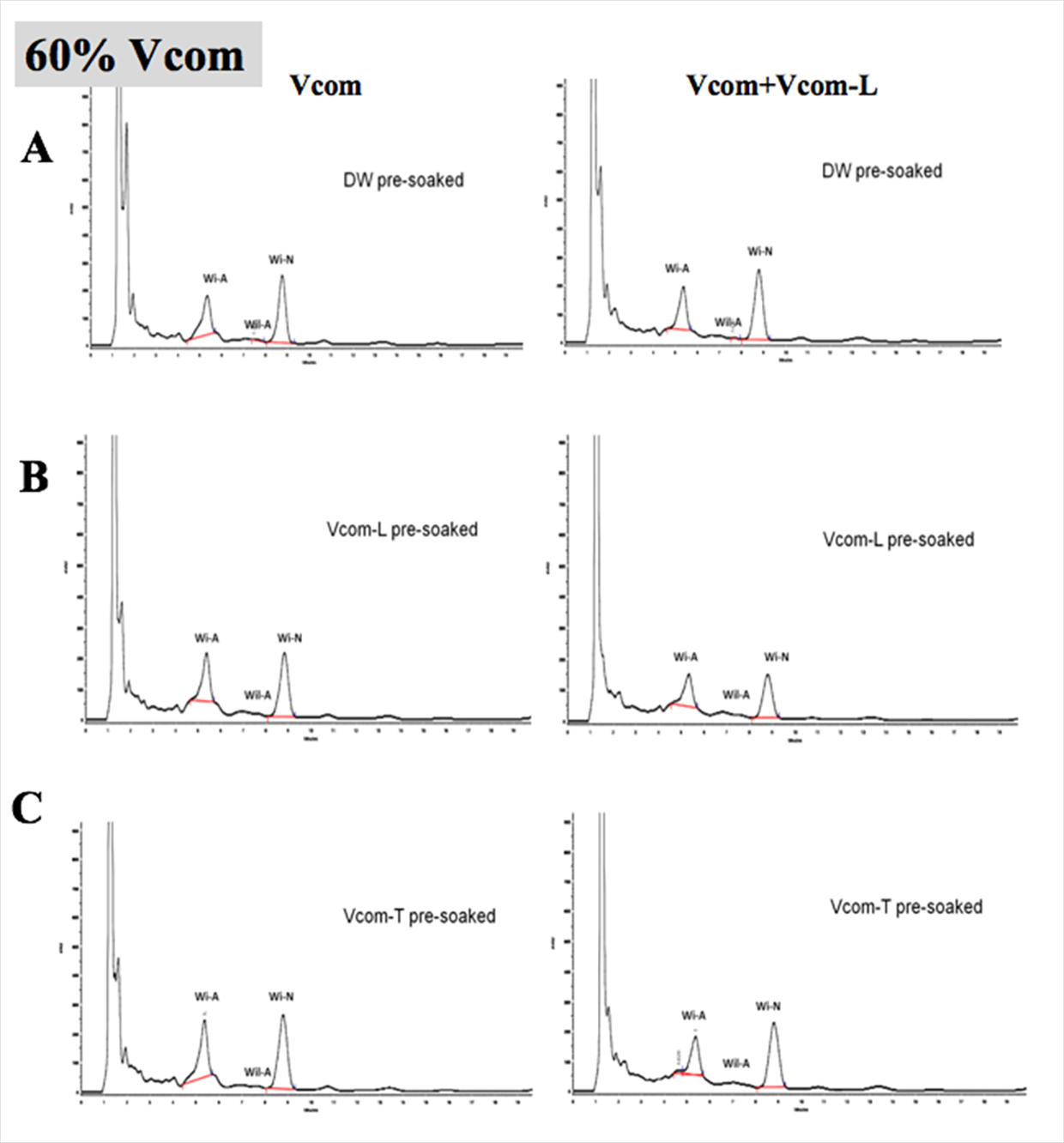

Supplement: S5 Fig — (TIF) [file pone.0194314.s005.tif]
